# Identification of potassium phosphite responsive miRNAs and their targets in potato

**DOI:** 10.1371/journal.pone.0222346

**Published:** 2019-09-12

**Authors:** María Florencia Rey-Burusco, Gustavo Raúl Daleo, Mariana Laura Feldman

**Affiliations:** Instituto de Investigaciones Biológicas-CONICET, Facultad de Ciencias Exactas y Naturales, Universidad Nacional de Mar del Plata, Mar del Plata, Buenos Aires, Argentina; Kunming University of Science and Technology, CHINA

## Abstract

Micro RNAs (miRNAs) are small single strand non-coding RNAs that regulate gene expression at the post-transcriptional level, either by translational inhibition or mRNA degradation based on the extent of complementarity between the miRNA and its target mRNAs. Potato (*Solanum tuberosum* L.) is the most important horticultural crop in Argentina. Achieving an integrated control of diseases is crucial for this crop, where frequent agrochemical applications, particularly fungicides, are carried out. A promising strategy is based on promoting induced resistance through the application of environmentally friendly compounds such as phosphites, inorganic salts of phosphorous acid. The use of phosphites in disease control management has proven to be effective. Although the mechanisms underlying their effect remain unclear, we postulated that miRNAs could be involved. Therefore we performed next generation sequencing (NGS) in potato leaves treated and non treated with potassium phosphite (KPhi). We identified 25 miRNAs that were expressed differentially, 14 already annotated in miRBase and 11 mapped to the potato genome as potential new miRNAs. A prediction of miRNA targets showed genes related to pathogen resistance, transcription factors, and oxidative stress. We also analyzed *in silico* stress and phytohormone responsive cis-acting elements on differentially expressed pre miRNAs. Despite the fact that some of the differentially expressed miRNAs have been already identified, this is to our knowledge the first report identifying miRNAs responsive to a biocompatible stress resistance inducer such as potassium phosphite, in plants. Further characterization of these miRNAs and their target genes might help to elucidate the molecular mechanisms underlying KPhi-induced resistance.

## Introduction

Potato (*Solanum tuberosum* L.) is the fourth food crop worldwide and the most important horticultural crop in Argentina, where 80,000 hectares of potatoes are planted per year, mainly in the provinces of Córdoba and Buenos Aires [1]. Integrated control of diseases is crucial for this crop, where frequent agrochemical applications, particularly fungicides, are carried out to manage late blight disease caused by *Phytophthora infestans* [2,3]. An alternative strategy is based on promoting induced resistance through the application of environmentally friendly compounds. In this context, phosphites have been extensively used as biocompatible inducers of defense responses [4].

The role of phosphites in disease control management has been studied extensively, and previous results have shown many promising properties associated with these compounds: the stimulation of plant defense mechanisms such as enhanced production of reactive oxygen species (ROS), the induction of pathogenesis related proteins (PRs) and the reinforcement of the cell wall [5–7]. It has also been demonstrated that KPhi primes an intense and rapid response to infection, involving heightened activation of a range of defense responses [8–10]. Additionally, KPhi has been proven to be an effective protective agent against UV-B stress [11]. However the mechanisms underlying KPhi protective effect remain unclear, the complexity of defense mechanisms and the plethora of pathways involved, suggest that miRNAs might be involved. miRNAs can target and subsequently regulate the expression of multiple genes simultaneously, providing a rapid and efficient way of regulating plant responses to stress.

miRNAs are small single-stranded, non-coding RNAs present both in animals and plants, that regulate gene expression at the post-transcriptional level, either by repressing mRNA translation or mediating the degradation of the targeted mRNAs depending on their degree of complementarity [12]. Specifically in plants, this class of ~22 nt RNAs play crucial roles in plant biological processes and responses to disease and environmental stresses [13–15]. Many plant miRNAs and their targets have been identified by computational and experimental approaches, in various species [16,17]. Several conserved miRNA families have been described in potato, some of them targeting transcription factors with diverse roles in plant growth and development, genes involved in signal transduction, and hormone signaling pathways. miRNA families such as mir172, miR156, miR164, miR166, miR167, miR171, miR390, miR394, miR395, miR399, miR530, miR829, mir395, mir398, miR414, miR778, among others, appear to be involved in potato response to various disease and environmental stresses [18–20].

We have hypothesized that one or more miRNAs might control the key-steps that lead to defense reactions mediated by KPhi in potato. In this scenario, the present work aims to identify miRNAs involved in the regulation of potato defense responses after potassium phosphite treatment.

## Materials and methods

### Plant material and phosphite treatment

*S*. *tuberosum* seed tubers (cv. Shepody) were planted in pots containing a pasteurized mixture of soil and vermiculite (3:1, v/v). Pots were maintained under greenhouse conditions (18°C day-night temperature, 16 h of light per day). Potassium phosphite (KPhi), 1% (v/v) water solution of the commercial product (Afital Potassium Phosphite, Agro-EMCODI SA) was applied to the foliage at 5 mL per plant (3 L/ha) by using an atomizer (ESAC SA) operating at 200 kPa, 21 days after emergence. Control plants were sprayed with distilled water. Leaf tissue was collected after 72 hs of KPhi or water treatment. Experiments were performed at least three times for each condition.

### RNA isolation

Total RNA from each treatment was isolated from 0.1 g of fresh leaf tissue using the RNeasy kit (Qiagen), following the manufacturer’s protocol. The purified RNAs were analyzed using a Thermo Scientific NanoDropOne spectrophotometer. RNA integrity was checked by 1% agarose gel electrophoresis. Three biological replicates were performed for each condition: control (C) and KPhi treated (T).

### Next generation sequencing

Total RNA (1 ug) was used to prepare small RNA libraries according to the TruSeq Small RNA Sample Prep Kits protocol (Illumina, San Diego, USA). Purified cDNA libraries were 50bp single-end sequenced on an Illumina Hiseq 2500 at LC Sciences (Houston, Texas, USA) following the vendor's recommended protocol.

### Identification of known and novel miRNAs

**Fig 1:**
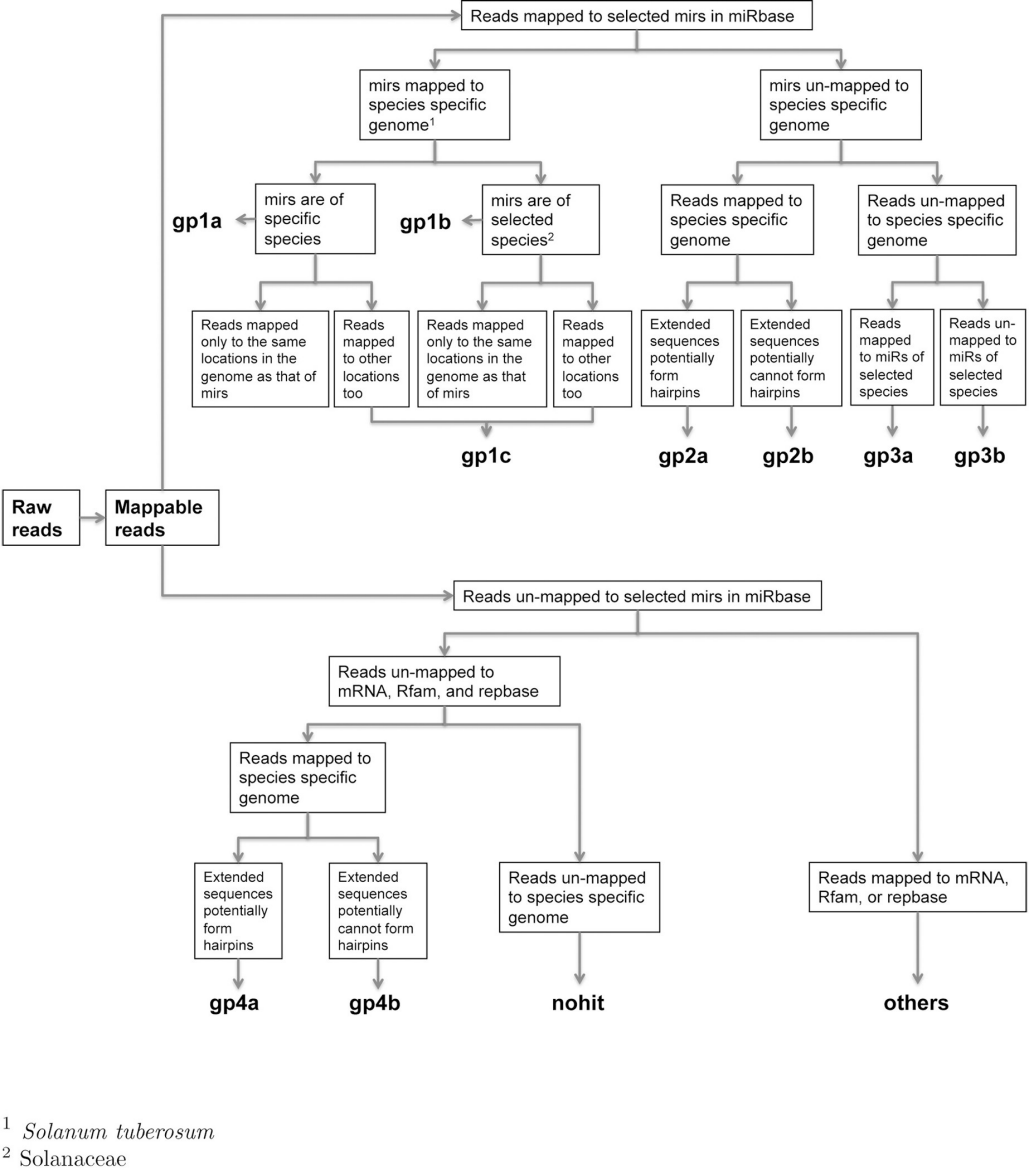
Flow chart of data analysis.

Raw reads were subjected to an in-house program, ACGT101-miR (LC Sciences, Houston, Texas, USA) to remove adapter dimers, contaminating sequences with no 3’ adapters (3ADT), low complexity regions, common RNA families (rRNA, tRNA, snRNA, snoRNA) and repeats. Subsequently, unique sequences with 18~25 nucleotides length were mapped to *Solanum tuberosum* (specific species) precursors in miRBase 21.0 by BLAST search to identify known miRNAs and novel 3p- and 5p- derived miRNAs. Length variation at both 3’ and 5’ ends and one mismatch inside of the sequence were allowed in the alignment. The unique sequences mapping to specific species mature miRNAs in hairpin arms were identified as known miRNAs. The unique sequences mapping to the other arm of known specific species precursor hairpin opposite to the annotated mature miRNA-containing arm were considered to be novel 5p- or 3p derived miRNA candidates. The remaining sequences were mapped to *Solanum lycopersicum* precursors (selected species of *Solanaceae* family) in miRBase 21.0 by BLAST search, and the mapped pre-miRNAs were further BLASTed against the specific species genomes to determine their genomic locations. The above two were defined as known miRNAs. The unmapped sequences were BLASTed against the specific genomes, and the hairpin RNA structures containing sequences were predicted from the flank 120 nt sequences using RNAfold software (http://rna.tbi.univie.ac.at/cgi-bin/RNAWebSuite/RNAfold.cgi). According to the mapping performed and secondary structure prediction reads were assigned to different groups (as described in detail in Fig 1).

The naming system of miRNAs identified in this study is as follows: the miRNA name is composed of the first known miR name in a cluster, an underscore, and a matching annotation: L–n means the miRNA_seq (detected) is n base less than known rep_miRSeq in the left side; R–n means the miRNA_seq (detected) is n base less than known rep_miRSeq in the right side; L+n means the miRNA_seq (detected) is n base more than known rep_miRSeq in the left side; R+n means the miRNA_seq (detected) is n base more than known rep_miRSeq in the right side; 1ss8GA means 1 substitution (ss), which is G to A at position 8. If there is no matching annotation, the miRNA_seq (detected) is exactly the same as known rep_miRSEq. miRNAs located on the other arms of hairpin structures are annotated as p5/p3 to distinguish from the reported 5/3 sequences.

### Differential expression analysis

Differential expression of miRNAs based on normalized deep-sequencing counts was analyzed using Student's t-test. The significance threshold was set to 0.01 and 0.05 in each test. Sequence reads of the six libraries were normalized to 1 million by the total number of clean small RNA reads in each sample. The log_2_ ratio formula was as follows: log_2_ ratio = log_2_ (miRNA reads in KPhi treated/miRNA reads in control).

### Target prediction and GO analysis

Target predictions were performed with psRNATarget web server (http://plantgrn.noble.org/psRNATarget/analysis) using default parameters with a maximum of 3 expectation cut-off [21]. To better understand their function, the putative target genes of the differentially expressed miRNAs were subjected to GO analysis. Significant GO terms were calculated by a Hypergeometric equation, and those GO terms with p-value<0.05 were defined as significant.

### qRT-PCR analysis of miRNAs and their predicted targets

The expression level of miRNAs was detected by stem-loop based reverse transcription and quantitative real time PCR (qRT-PCR) [22]. miRNAs and mRNAs were reverse transcribed to cDNAs with stem-loop specific primers and oligo dT primers, respectively, using the GoScript (TM) Reverse Transcriptase (Promega). cDNA was generated from 1ug of total RNA, with both miRNA specific stem-loop primers and oligo dT primers in each reaction tube at 16°C for 30 minutes, 42°C for 30 minutes, 50°C for 60 minutes and 70°C for 15 minutes.

The expression levels of miRNAs and mRNAs were analyzed by quantitative real-time PCR (qPCR) using an Applied Biosystems StepOneTM Plus Real-Time PCR System (Applied Biosystems, Waltham, MA, United States) and FastStart Universal SYBR Green Master mix (Roche). For miRNAs, samples were incubated at 95°C for 5 minutes, and the amplification was set at 45 cycles of denaturation at 95°C for 15 seconds, annealing at 60°C for 30 seconds, and an extension step at 72°C for 20 seconds. For miRNA target genes, samples were incubated at 95°C for 10 minutes, and amplification was set at 40 cycles of denaturation at 95°C for 15 seconds and an annealing step at 60°C for 1 minute. miRNA and target-gene specific primers were designed using NGS data and available databases (http://www.mirbase.org/, http://solgenomics.net/) (S1 Table). *EF1α* was employed as an internal control for mRNAs and miRNAs. Reactions were performed with three biological replicates and relative gene expression level was analyzed using the comparative 2^ΔΔ^CT method [23]. Pair-wise fixed reallocation randomization test statistical analysis was performed with REST software (Relative Expression Software Tool) [24].

### Analysis of cis-acting elements

The presence of well characterized cis-acting elements in the promoters of pre-miRNAs was analyzed *in silico* using the web available software PlantCARE (http://bioinformatics.psb.ugent.be/webtools/plantcare/html/search_CARE.html) [25]. Upstream sequences of potato pre-miRNAs (1000 bp) were retrieved from the PGSC v4.03 database (http://solanaceae.plantbiology.msu.edu/).

### Availability of supporting data

The data supporting this work are available in the public database: Gene Expression Omnibus (GEO), under the accession number GSE132232.

## Results

### High throughput sequencing of small RNAs

Total RNA from potassium phosphite treated (T2, T4, T6) and non treated leaves (C1, C3, C5) was isolated, and six small RNA libraries were constructed to perform high throughput sequencing. RNAseq from the libraries generated 58,391,355 total raw reads. In particular, C1 library yielded 8,599,726 reads, while C3 and C5 libraries yielded 8,051,010 and 8,175,531 reads, respectively. T2, T4, and T6 yielded 12,864,315, 10,563,970 and 10,136,803 reads, respectively (S2 Table).

**Fig 2:**
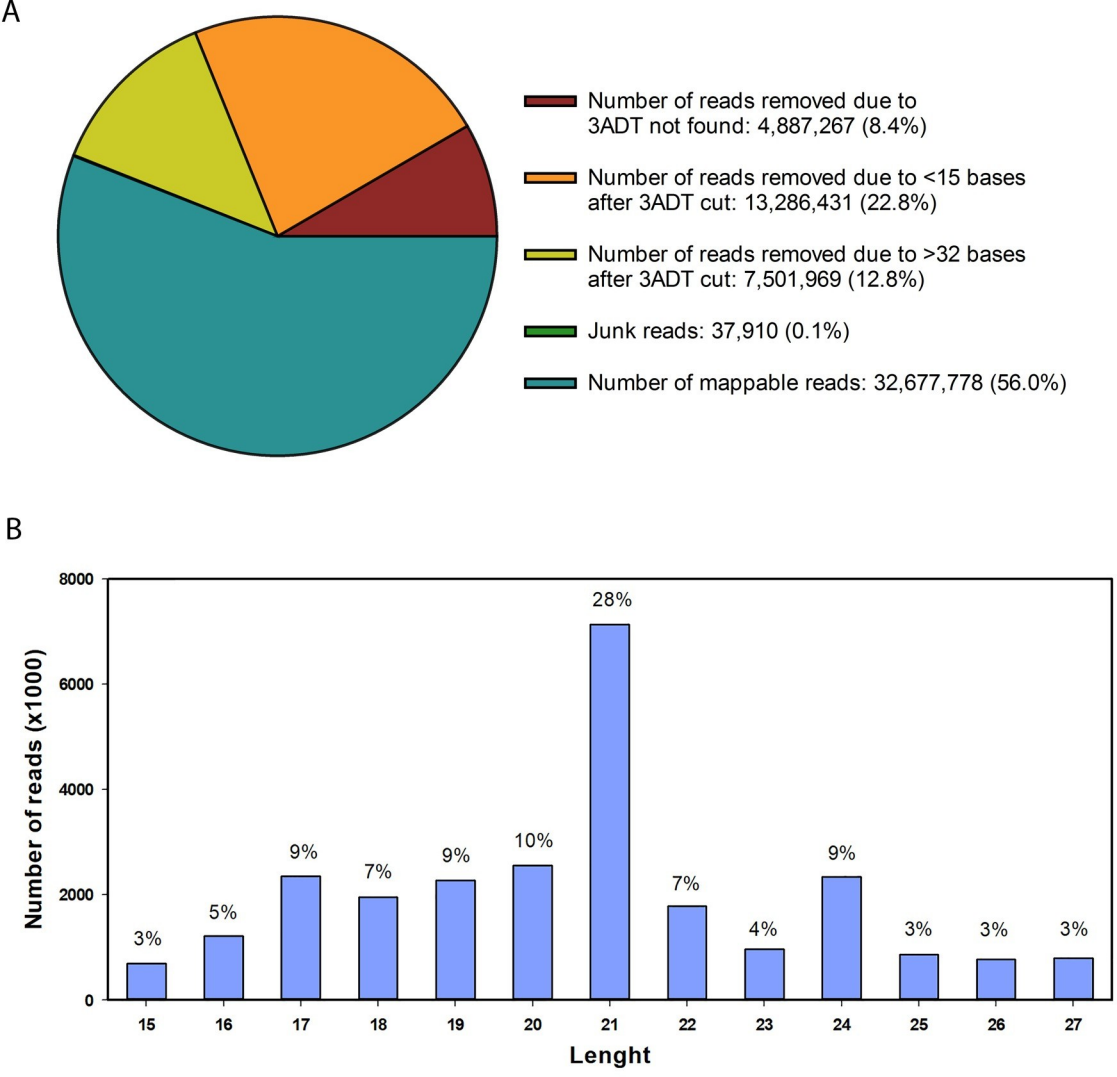
Data filtering and length distribution of mappable reads. Raw reads were filtered to remove sequences with no 3’ adapters (3ADT), junk reads and reads shorter than 15 and longer than 32 nucleotides; and were subsequently mapped to specific and selected genomes. (A) Pie plot of data filtering. (B) Length distribution of mappable reads.

After low-quality reads, adapters, poly A sequences and short RNAs shorter than 15 nucleotides were removed, 32,677,778 unique small RNA reads (56% of the total raw reads) remained (Fig 2A). RNA length ranged between 15–32 nt, with the highest proportion corresponding to 21 nt (Fig 2B). Reliable reads were divided into 4 groups as described in Fig 1. A total of 206 *S*. *tuberosum* known miRNAs (group 1a) were identified along with additional 42 *Solanaceae* miRNAs (group 1b), novel to potato. Interestingly, 388 reads that mapped to the potato genome, presented extended sequences that potentially form hairpins constituting potential novel miRNAs (group 4a) (Table 1).

**Table 1:** Summary of data analysis results.

		Group	# of sequences	# of unique miRNAs
	Raw		58,391,355	
	Total mappable reads		32,677,778	
**Known miRNAs**	of specific species^1^	1a	3,506,752	206
	of selected species^2^, but novel to specific species Group	1b	113,297	42
**Predicted miRNAs**	Mapped to known miRNAs of selected species and genome; within hairpins	2a	1,515,585	38
	Mapped to known miRNAs of selected species and genome; no hairpins	2b	871	132
	Mapped to known miRNAs and miRNAs of selected species but unmapped to genome	3a	1,230	52
	Mapped to known miRNAs of selected species but unmapped to genome	3b	550	52
	Unmapped to known miRNAs but mapped to genome and within hairpins	4a	185,614	388
	Overall			910
	Others (mapped to mRNAs, RFam, or repbase)		7,554,022	
	Nohit		7,916,889	

^1^ *Solanum tuberosum*

^2^ *Solanaceae*

### Differential expression analysis of known and novel miRNAs after KPhi treatment

**Fig 3:**
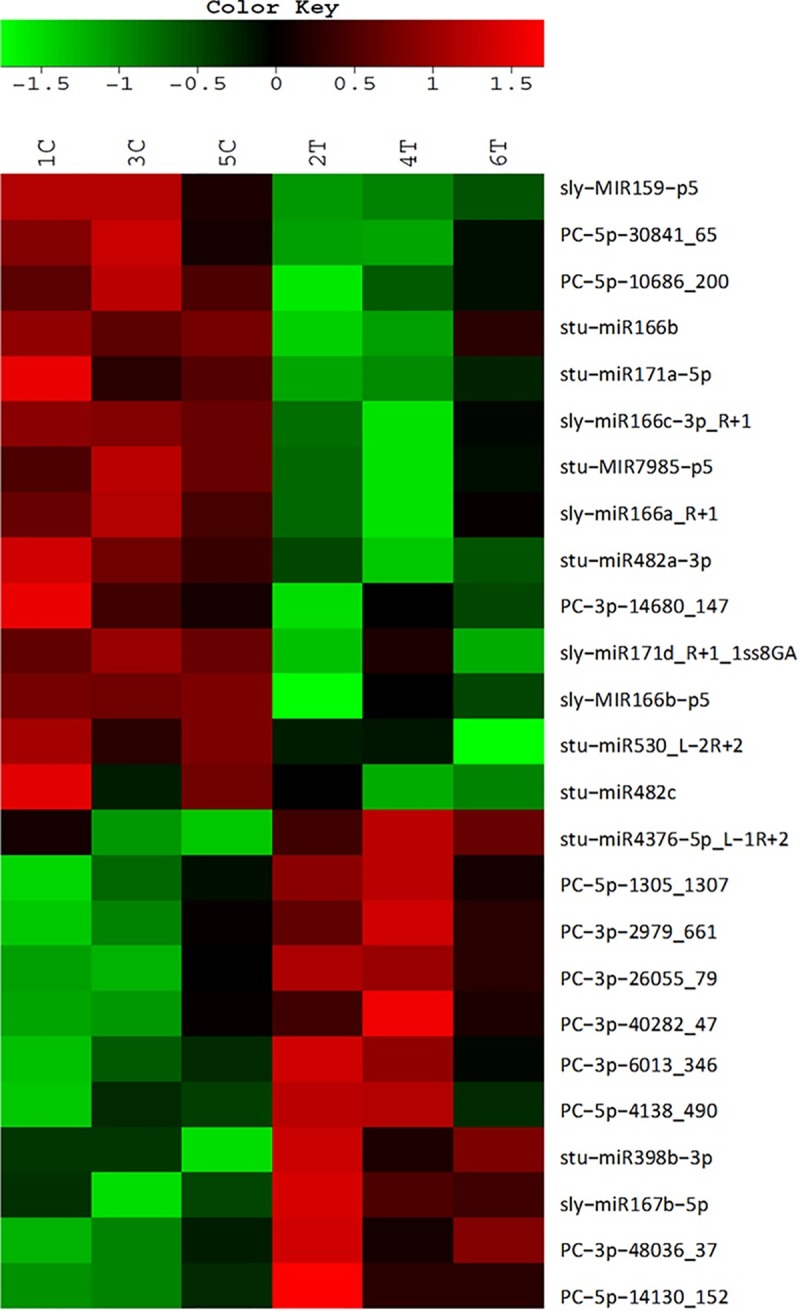
Heat map of differentially expressed miRNAs. KPhi treated (T2, T4, T6) and non treated (C1, C3, C5) replicates were analyzed. Differential expression of miRNAs based on normalized deep-sequencing counts, was analyzed using a Student's t-test. Up-regulated and down-regulated miRNAs are represented in red and green, respectively.

To identify differentially expressed miRNAs after KPhi treatment, six small RNA libraries were constructed from treated and non treated plants and sequenced independently. Statistical analysis showed that 25 miRNAs were differentially expressed after KPhi treatment (Fig 3, S3 Table). Among them, 14 were already annotated in the miRBase, and 11 were mapped to the potato genome as potential novel miRNAs (PCs). From these 14 known miRNAs, 3 were up-regulated (stu-miR398b-3p, sly-miR167b-5p and stu-miR4376-5p L-1R+2) and 11 were down-regulated (stu-miR166b, stu-miR482c, stu-miR482a-3p, sly-miR159-p5, stu-miR171a-5p, stu-MIR7985-p5, sly-miR166c-3p_R+1, sly-miR166a_R+1, sly-miR171d_R+1_1ss8GA, stu-miR530_L-2R+2, sly-MIR166b-p5).

Among the 11 potentially new miRNAs, 8 were up regulated (PC-3p-48036_37, PC-3p-26055_79, PC-5p-1305_1307, PC-3p-2979_661, PC-3p-6013_346, PC-3p-40282_47, PC-5p-14130_152, PC-5p-4138_490) and 3 were down regulated (PC-5p-30841_65, PC-5p-10686_200, PC-3p-14680_147).

**Fig 4:**
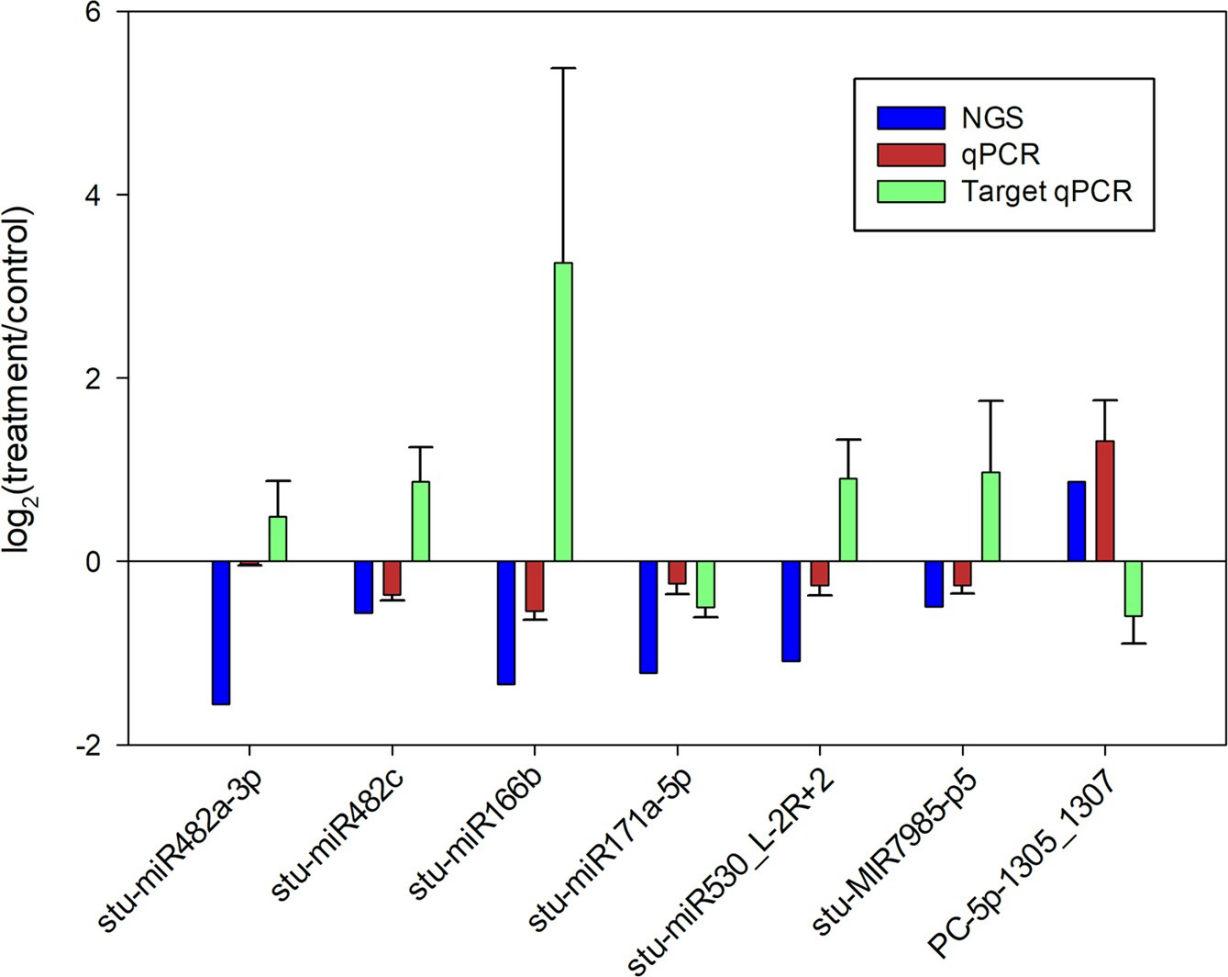
Validation of next generation sequencing (NGS) results for selected differentially expressed miRNAs and analysis of selected target genes. Quantitative real time PCR (qPCR) was performed to validate differentially expressed miRNAs (stu-miR482a-3p, stu-miR482c, stu-miR166b, stu-miR171a-5p, stu-miR530_L-2R+2, stu-MIR7985-p5, PC-5p-1305_1307) and targets (TIR-NBS-LRR resistance protein, Resistance protein PSH-RGH7, BZIP domain class transcription factor, Methylketone synthase Ib, Zinc knuckle (CCHC-type) family protein, Spotted leaf protein, F-box protein family). The EF1*α* gene was used as housekeeping. Three biological replicates were employed. Vertical bars represent the standard deviation of the mean (n  =  3).

To confirm the high-throughput sequencing data, stem loop qRT-PCR was performed for 7 of the differentially expressed miRNAs. A comparison of the results from qPCR with those from NGS revealed similar patterns of expression (Fig 4).

### Target prediction and GO analysis

To elucidate the biological functions of KPhi differentially expressed miRNAs, mRNA sequence complementarity was assessed using the psRNATarget software. A total of 211 potential target genes were identified for the 14 conserved miRNAs, based on their perfect or near-perfect complementarity to their target mRNA sequences. For most of the differentially expressed miRNAs, more than one potential target gene was predicted. Additionally, several target genes (a total of 118) were identified for most of the potentially novel miRNAs. Most of the predicted targets belong to transcription factor gene families, such as bZip, GRAS, AP2, REV HD-ZipIII, ARF, among others. Other miRNAs were predicted to target genes involved in the regulation of plant metabolism and stress responses. Detailed annotations of these results are presented in S4 Table.

Some of the miRNA predicted targets were analyzed by qPCR (Fig 4, Table 2). These results showed that 6 of the analyzed targets followed a negatively correlated expression pattern with their miRNAs. However, miRNA171a-5p target (methylketone synthase Ib) expression, was not consistent with its miRNA down-regulation (Fig 4).

**Table 2:** Selected differentially expressed miRNAs after KPhi treatment and targets.

miRNA	Differential expression under KPhi treatment	Target accession	Target description
**stu-miR482a-3p**	Down-regulation	PGSC0003DMT400053047	TIR-NBS-LRR resistance protein
**stu-miR482c**	Down-regulation	PGSC0003DMT400012486	Resistance protein PSH-RGH7
**stu-miR166b**	Down-regulation	PGSC0003DMT400074934	BZIP domain class transcription factor
**stu-miR171a-5p**	Down-regulation	PGSC0003DMT400066685	Methylketone synthase Ib
**stu-miR530_L-2R+2**	Down-regulation	PGSC0003DMT400002883	Zinc knuckle (CCHC-type) family protein
**stu-MIR7985-p5**	Down-regulation	PGSC0003DMT400062931	Spotted leaf protein
**PC-5p-1305_1307**	Up-regulation	PGSC0003DMT400027717	F-box family protein

**Fig 5:**
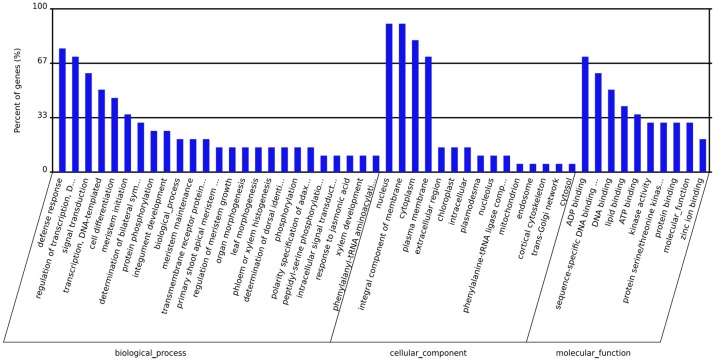
Gene ontology analysis of differentially expressed miRNA targets. All identified target genes were classified according to their Biological process (BP), Molecular function (MF), and Cellular component (CC) based on GO enrichment analysis. Statistical significance was set to p<0.05.

To further analyze the biological function of miRNA targets, GO analysis was performed. The highest percentage of genes falls into defense response, regulation of transcription, and signal transduction categories (biological process group). Cellular components and molecular functions of most of the genes are consistent with their biological process group (Fig 5, S5 Table)

### *In-silico* analysis of cis-elements present in KPhi responsive miRNA promoters

**Fig 6:**
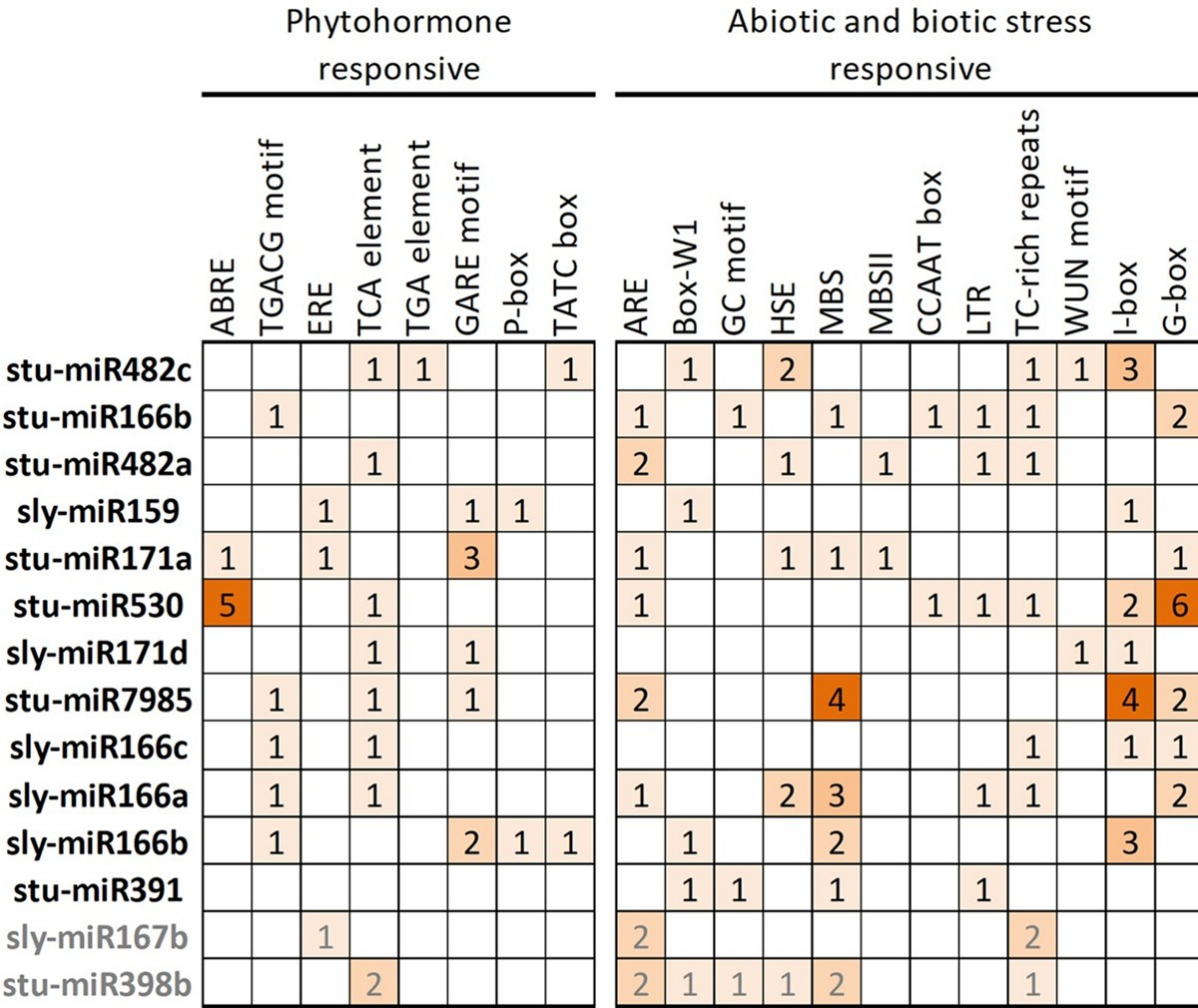
*In silico* identification of cis-elements in the promoters of KPhi differentially expressed miRNAs. The promoters of pre-miRNAs were analyzed *in silico* using the web available program PlantCARE (bioinformatics.psb.ugent.be/webtools/plantcare/html). Down-regulated and up-regulated miRNAs are shown in black and grey, respectively. The numbers represent total well characterized cis-acting elements per miRNA.

Several stress responsive cis-elements were identified *in-silico* in promoters of potassium phosphite differentially expressed miRNAs (Fig 6): anaerobic response element (ARE), element sensitive to the fungal inducer (Box-W1), MYB binding site related to the abiotic stress (MBS) and its stress-related binding site MYBHv1 (CCAAT-box), MYB binding site involved in the regulation of flavonoid biosynthetic genes (MBSII), enhancer element involved in specific anoxic inducibility (GC-motif), cis-acting element involved in heat stress response (HSE), low temperature response element (LTR), repetitions rich in TC involved in defense and response to stress (TC-reach repeats), wound-sensitive element (WUN), UV light stress related cis-elements (box I and box G). All miRNAs presented more than one stress related cis-elements in their promoter regions, suggesting they might participate in multiple stress response signaling pathways, and some of them were in multiple copy numbers. In addition to stress-related elements, most of the KPhi differentially expressed miRNAs presented motifs associated to phytohormones: element sensitive to abscisic acid (ABRE), cis-acting regulatory element involved in the methyl jasmonic acid response (TGACG-motif), ethylene sensitive element (ERE), cis-acting element involved in the response to salicylic acid (TCA element), auxin-sensitive element (TGA element), cis-acting elements involved gibberellin response (GARE motif, P-box and TATC box).

## Discussion

Despite the available information about the stress protective effect of KPhi on different crops, the mode of action is unclear and the target/effector molecules in the plant are still unknown. The present study aimed to gain an insight into the complex mechanisms of action of KPhi. To achieve this objective, we performed a sequencing analysis to determine changes in miRNA expression and analyzed their targets in potato leaves treated with KPhi. Additionally, to identify common cis-elements involved in miRNA regulation, we analyzed their promoters *in-silico*.

High-throughput sequencing results revealed the presence of 206 unique miRNAs belonging to potato, and 42 unique miRNAs from tomato, but not previously described in potato. Additionally, a total of 288 potential new miRNAs were identified. Differential expression analysis showed changes in the relative abundance of 25 miRNAs after KPhi treatment, 14 of them belonging to known *Solanaceae* miRNA families and 11 of them being potential novel miRNAs. qPCR results were consistent with NGS differential expression analysis for 7 miRNAs (stu-miR482a-3p, stu-miR482c, stu-miR166b, stu-miR171a-5p, stu-miR530_L-2R+2, stu-MIR7985-p5, PC-5p-1305_1307).

The analysis of putative target genes of miRNAs responsive to KPhi treatment gave an insight into the potential mechanisms and effectors of this compound. In this context, we analyzed the expression of 7 target genes: TIR-NBS-LRR resistance protein, Resistance protein PSH-RGH7, BZIP domain class transcription factor, Methylketone synthase Ib, Zinc knuckle (CCHC-type) family protein, Spotted leaf protein and, F-box family protein (Table 2). qPCR results showed that these genes were negatively correlated with the expression pattern of their complementary miRNA, with the exception of the putative target of miR171a-5p (methylketone synthase Ib). The unexpected behavior of the analyzed methylketone synthase gene might indicate that other regulatory mechanisms prevail over miR171 regulation, in the conditions of the present study (KPhi treatment), or that methylketone synthase is not a miRNA171 target in this system thus, miR171 might exert its action through other untested target genes.

The target genes analyzed in this work are mainly involved in plant defense mechanisms. For instance, it is known that bZIP transcription factors (miR166b targets) are involved in both abiotic and biotic stress responses [26]. Zhou *et al*. (2018) have described a potato bZIP transcription factor (StbZIP61) that regulates the biosynthesis of salicylic acid (SA) in the defense response against *P*. *infestans* [27]. Moreover, putative targets of miR482a-3p and miR482c PSH-RGH7 resistance and TIR-NBS-LRR resistance proteins, respectively, belong to the class of characterized disease resistance genes (R genes). Plant R genes encode nucleotide-binding site leucine-rich repeat (NBS-LRR) proteins postulated to be involved in the detection of diverse specialized effectors from pathogens such as bacteria, viruses, fungi, nematodes, insects and oomycetes [28–31]. Despite the fact that in various plant species, computational analyses predicted a high number of genes regulated by miRNAs, a few of them were validated in potato. In this work, we validated in potato, one of the predicted targets genes for stu-miR530_L-2R+2, a Zinc Knuckle (CCHC-type) family protein. Zinc Knuckle are zinc finger proteins containing a characteristic motif with two short β-strands joined by a turn. The expression of a potato zinc finger protein gene, StZFP1, has been reported to increase upon biotic and abiotic stress, and after exogenous ABA application [32]. Although miR530 has been shown to target a zinc knuckle protein in rice [33], there are no previous reports available in potato. stu-MIR7985-p5 was differentially expressed after KPhi treatment and targeted a Spotted Leaf protein (SLp). Spotted Leaf proteins are U-box domain proteins that have been described to participate in the regulation of cell death and defense response mechanisms [34,35]. Zhang *et al*. (2013) have identified miR7985 in *Solanum tuberosum* by high-throughput sequencing [18], however, we report for the first time that this miRNA targets an SLp protein in potato.

We have validated the differential expression of PC-5p-1305_1307, a putative novel miRNA that was up-regulated after KPhi treatment. This potential miRNA targets, and negatively regulates an F-box protein. F-box proteins are key factors in phytohormone perception and stress signaling, photoperiodism, and metabolism regulation [36]. Our results are in accordance with previous works that have reported, in Arabidopsis plants exposed to UV-B radiation, the suppressed expression of 4 F-box proteins, resulting in enhanced polyphenol levels, suggesting that they are involved in tolerance mechanisms against UV-B abiotic stress [37].

Interestingly, most of the predicted target genes for the potential novel miRNAs described in this work belong to genes involved in plant defense reactions (S4 Table) and might play an important role in the regulation of the molecular events mediated by KPhi. Further experiments such as the validation of the potential new miRNAs and, a degradome analysis must be performed to analyze the battery of target genes that are differentially expressed after KPhi treatment.

In order to gain information about the possible regulation of KPhi responsive miRNAs, the presence of well characterized cis-acting elements in the promoters of pre-miRNAs was analyzed *in-silico*. We focused on cis-elements involved in biotic and abiotic stress and phytohormone responsiveness. Noteworthy, cis-elements that respond to phytohormones, fungal elicitors and stress were found in the promoters of most pre-miRNAs. A few stress-related cis-elements were found in most of KPhi responsive miRNAs (ARE, MBS, TC rich repeat, I box, and G-box). Regarding phytohormone responsiveness, TCA, a cis-element responsive to phytohormones, was present in most differentially expressed miRNAs. Interestingly, stu-miR530_L-2R+2 has 8 light-responsive elements (G-box and I-box) and 5 ABA-regulated sites. stu-MIR7985-p5 has 4 abscisic acid inducible cis-elements (MBS), while miRNAs stu-miR166b, sly-miR159, stu-miR482c, stu-miR398b, and sly-miR167b have a Box-W1, a motif responsive to, a motif responsive to fungal elicitors and wounding.

The results presented in this work are consistent with the protective function of KPhi in potato described previously elsewhere. The diversity of differentially expressed miRNAs and the variety of cis-elements present in their promoters suggest that miRNAs are likely to participate in KPhi-dependent induction of a plethora of defense pathways [5–7,11,38–40]. It has also been reported that these responses are dependent on the action of phytohormones such as SA, jasmonic acid (JA), auxins, and ethylene [10,41,42]. The cross-talk between hormone signaling pathways in plants has been extensively documented [43,44]. In this scenario, phosphites might be a part of the intricate network of hormones/effectors activated as a defensive response upon stress.

In summary, miRNAs that respond to KPhi treatment have a wide range of functions in plants, as their predicted target genes. These results are consistent with the number and diversity of responses that KPhi triggers in potato. In this work, we provide evidence that the amplitude of responses associated with KPhi treatment can be, at least in part, explained by the diversity of miRNAs that are differentially expressed.

This is to our knowledge the first analysis of responsive miRNAs to a biocompatible stress resistance inducer as KPhi in potato. Additionally, we validated for the first time two predicted targets for potato miRNAs and a potential novel miRNA. Further characterization of these miRNAs and their target genes, might help to elucidate the molecular mechanisms underlying KPhi-induced resistance. This might in turn, aid in the design of genetically engineered potatoes to achieve a product with enhanced resistance to environmental stress.

## Supporting information

S1 Table miRNAs and target genes primers. (DOC)

S2 Table RNA seq library reads. (XLS)

S3 Table miRNAs differential expression. (XLS)

S4 Table Target prediction. (XLS)

S5 Table GO analysis. (XLS)

## References

[1] FAO STAT Food and Agriculture Organization: Crops statistics database. FAO Prod Stat. 2017 www.fao.org/faostat

[2] Arora RK, Sharma S, Singh BP. Late blight disease of potato and its management. Potato J. 2014; 4: 16–40.

[3] Importance of potato late blight in Argentina, and the effect of fungicide treatments on yield increments over twenty years. Cien Inv Agr. 2009;36: 115–122. 10.4067/S0718-16202009000100011

[4] Trejo-Téllez LI, Gómez-Merino FC. Phosphite as an inductor of adaptive responses to stress and stimulator of better plant performance In: Vats S, editor. Biotic and Abiotic Stress Tolerance in Plants. Springer, Singapore 2018 10.1007/978-981-10-9029-5_8

[5] Lobato MC, Olivieri FP, Altamiranda EAG, Wolski EA, Daleo GR, Caldiz DO, et al Phosphite compounds reduce disease severity in potato seed tubers and foliage. Eur J Plant Pathol. 2008;122: 349–358. 10.1007/s10658-008-9299-9

[6] Lobato MC, Machinandiarena MF, Tambascio C, Dosio GAA, Caldiz DO, Daleo GR, et al Effect of foliar applications of phosphite on post-harvest potato tubers. Eur J Plant Pathol. 2011;130: 155–163. 10.1007/s10658-011-9741-2

[7] Olivieri FP, Feldman ML, Machinandiarena MF, Lobato MC, Caldiz DO, Daleo GR, et al Phosphite applications induce molecular modifications in potato tuber periderm and cortex that enhance resistance to pathogens. Crop Prot. 2012;32: 1–6. 10.1016/j.cropro.2011.08.025

[8] Burra DD, Berkowitz O, Hedley PE, Morris J, Resjö S, Levander F, et al Phosphite-induced changes of the transcriptome and secretome in *Solanum tuberosum* leading to resistance against *Phytophthora infestans*. BMC Plant Biol. 2014;14: 1–17. 10.1186/1471-2229-14-1 25270759 PMC4192290

[9] Eshraghi L, Anderson J, Aryamanesh N, Shearer B, Mccomb J, Hardy GESJ, et al Phosphite primed defense responses and enhanced expression of defense genes in *Arabidopsis* thaliana infected with *Phytophthora cinnamomi*. Plant Pathol. 2011;60: 1086–1095. 10.1111/j.1365-3059.2011.02471.x

[10] Machinandiarena MF, Lobato MC, Feldman ML, Daleo GR, Andreu AB. Potassium phosphite primes defense responses in potato against *Phytophthora infestans*. J Plant Physiol. 2012;169: 1417–1424. 10.1016/j.jplph.2012.05.005 22727804

[11] Oyarburo NS, Machinandiarena MF, Feldman ML, Daleo GR, Andreu AB, Olivieri FP. Potassium phosphite increases tolerance to UV-B in potato. Plant Physiol Biochem. 2015;88: 1–8. 10.1016/j.plaphy.2015.01.003 25596554

[12] Dugas D V., Bartel B. MicroRNA regulation of gene expression in plants. Curr Opin Plant Biol. 2004;7: 512–520. 10.1016/j.pbi.2004.07.011 15337093

[13] Sunkar R. MicroRNAs with macro-effects on plant stress responses. Semin Cell Dev Biol. 2010;21: 805–811. 10.1016/j.semcdb.2010.04.001 20398781

[14] Jones-Rhoades MW, Bartel DP, Bartel B. MicroRNAS and their regulatory roles in plants. Annu Rev Plant Biol. 2006;57: 19–53. 10.1146/annurev.arplant.57.032905.105218 16669754

[15] Song X, Li Y, Cao X, Qi Y. microRNAs and their regulatory roles in plant-environment interactions. Annu Rev Plant Biol. 2019;70: 1–37. 10.1146/annurev-arplant-050718-100143 30848930

[16] Jones-Rhoades MW, Bartel DP. Computational identification of plant microRNAs and their targets, including a stress-induced miRNA. Mol Cell. 2004;14: 787–799. 10.1016/j.molcel.2004.05.027 15200956

[17] Devi KJ, Chakraborty S, Deb B, Rajwanshi R. Computational identification and functional annotation of microRNAs and their targets from expressed sequence tags (ESTs) and genome survey sequences (GSSs) of coffee (*Coffea arabica L*.). Plant Gene. 2016;6: 30–42. 10.1016/j.plgene.2016.03.001

[18] Zhang R, Marshall D, Bryan GJ, Hornyik C. Identification and characterization of miRNA transcriptome in potato by high-throughput sequencing. PLoS One. 2013;8 10.1371/journal.pone.0057233 23437348 PMC3578796

[19] Lakhotia N, Joshi G, Bhardwaj AR, Katiyar-Agarwal S, Agarwal M, Jagannath A, et al Identification and characterization of miRNAome in root, stem, leaf and tuber developmental stages of potato (*Solanum tuberosum L*.) by high-throughput sequencing. BMC Plant Biol. 2014;14: 1–16. 10.1186/1471-2229-14-1 24397411 PMC3913621

[20] Zhang W, Luo Y, Gong X, Zeng W, Li S. Computational identification of 48 potato microRNAs and their targets. Comput Biol Chem. 2009;33: 84–93. 10.1016/j.compbiolchem.2008.07.006 18723398

[21] Dai X, Zhao PX. PsRNATarget: A plant small RNA target analysis server. Nucleic Acids Res. 2011;39: 155–159. 10.1093/nar/gkq766 21622958 PMC3125753

[22] Varkonyi-Gasic E, Wu R, Wood M, Walton EF, Hellens RP. Protocol: A highly sensitive RT-PCR method for detection and quantification of microRNAs. Plant Methods. 2007;3: 1–12. 10.1186/1746-4811-3-1 17931426 PMC2225395

[23] Livak KJ, Schmittgen TD. Analysis of relative gene expression data using real time quantitative PCR and the 2∆∆C(T) Method. Methods. 2001;25(4): 402–408. 10.1006/meth.2001.1262 11846609

[24] Pfaffl MW, Horgan GW, Dempfle L. Relative expression software tool (REST) for group-wise comparison and statistical analysis of relative expression results in real-time PCR. Nucleic Acids Res. 2002;30: e36 10.1093/nar/30.9.e36 11972351 PMC113859

[25] Lescot M. PlantCARE, a database of plant cis-acting regulatory elements and a portal to tools for in silico analysis of promoter sequences. Nucleic Acids Res. 2002;30: 325–327. 10.1093/nar/30.1.325 11752327 PMC99092

[26] Alves MS, Dadalto SP, Gonçalves AB, De Souza GB, Barros VA, Fietto LG. Plant bZIP transcription factors responsive to pathogens: A review. Int J Mol Sci. 2013;14: 7815–7828. 10.3390/ijms14047815 23574941 PMC3645718

[27] Zhou XT, Jia LJ, Wang HY, Zhao P, Wang WY, Liu N, et al The potato transcription factor StbZIP61 regulates dynamic biosynthesis of salicylic acid in defense against Phytophthora infestans infection. Plant J. 2018;95: 1055–1068. 10.1111/tpj.14010 29952082

[28] Nandety RS, Caplan JL, Cavanaugh K, Perroud B, Wroblewski T, Michelmore RW, et al The role of TIR-NBS and TIR-X proteins in plant basal defense responses. Plant Physiol. 2013; 162: 1459–1472. 10.1104/pp.113.219162 23735504 PMC3707564

[29] Lee JJ, Park KW, Kwak YS, Ahn JY, Jung YH, Lee BH, et al Comparative proteomic study between tuberous roots of light orange- and purple-fleshed sweetpotato cultivars. Plant Sci. 2012;193–194: 120–129. 10.1016/j.plantsci.2012.06.003 22794925

[30] Slootweg E, Koropacka K, Roosien J, Dees R, Overmars H, Lankhorst RK, et al Sequence exchange between homologous NB-LRR genes converts virus resistance into nematode resistance, and vice versa. Plant Physiol. 2017;175: 498–510. 10.1104/pp.17.00485 28747428 PMC5580749

[31] Jiang N, Meng J, Cui J, Sun G, Luan Y. Function identification of miR482b, a negative regulator during tomato resistance to *Phytophthora infestans*. Hortic Res. Springer US. 2018; 5 10.1038/s41438-018-0017-2 29507733 PMC5830410

[32] Tian ZD, Zhang Y, Liu J, Xie CH. Novel potato C2H2-type zinc finger protein gene, StZFP1, which responds to biotic and abiotic stress, plays a role in salt tolerance. Plant Biology. 2010;12: 689–697. 10.1111/j.1438-8677.2009.00276.x 20701691

[33] Sun W, Xu XH, Wu X, Wang Y, Lu X, et al Genome-wide identification of microRNAs and their targets in wild type and phyB mutant provides a key link between microRNAs and the phyB-mediated light signaling pathway in rice. Front Plant Sci. 2015;6: 372 10.3389/fpls.2015.00372 26074936 PMC4448008

[34] Zeng LR, Qu S, Bordeos A, Yang C, Baraoidan M, Yan H, et al Spotted leaf11, a negative regulator of plant cell death and defense, encodes a U-Box/Armadillo repeat protein endowed with E3 ubiquitin ligase activity. Plant Cell Online. 2004;16: 2795–2808. 10.1105/tpc.104.025171 15377756 PMC520972

[35] Yamanouchi U, Yano M, Lin H, Ashikari M, Yamada K. A rice spotted leaf gene, Spl7, encodes a heat stress transcription factor protein. 2002;99: 7530–7535. 10.1073/pnas.112209199 PMC124274 12032317

[36] Zhang X, Gonzalez-Carranza ZH, Zhang S, Miao Y, Liu C-J, Roberts JA. F-Box proteins in plants. Annu Plant Rev. 2019; 1–21. 10.1002/9781119312994.apr0701

[37] Zhang X, Gou M, Guo C, Yang H, Liu C-J. Down-regulation of Kelch domain-containing F-Box protein in *Arabidopsis* enhances the production of (poly)phenols and tolerance to ultraviolet radiation. Plant Physiol. 2014;167: 337–350. 10.1104/pp.114.249136 25502410 PMC4326750

[38] Bengtsson T, Weighill D, Proux-Wéra E, Levander F, Resjö S, Burra DD, et al Proteomics and transcriptomics of the BABA-induced resistance response in potato using a novel functional annotation approach. BMC Genomics. 2014;15: 1–19. 10.1186/1471-2164-15-1 24773703 PMC4234511

[39] Lim S. Analysis of changes in the potato leaf proteome triggered by phosphite reveals functions associated with induced resistance against *Phytophthora infestans* Thesis. Dalhousie University. 2012; Available from: http://dalspace.library.dal.ca/handle/10222/15859

[40] Lim S, Borza T, Peters RD, Coffin RH, Al-Mughrabi KI, Pinto DM, et al Proteomics analysis suggests broad functional changes in potato leaves triggered by phosphites and a complex indirect mode of action against *Phytophthora infestans*. J Proteom. 2013;93: 207–23. 10.1016/j.jprot.2013.03.010 23542353

[41] Eshraghi L, Anderson JP, Aryamanesh N, McComb JA, Shearer B, Hardy GSJE. Suppression of the auxin response pathway enhances susceptibility to *Phytophthora cinnamomi* while phosphite-mediated resistance stimulates the auxin signalling pathway. BMC Plant Biol. 2014;14: 1–15. 10.1186/1471-2229-14-1 24649892 PMC3999932

[42] Massoud K, Barchietto T, Le Rudulier T, Pallandre L, Didierlaurent L, Garmier M, et al Dissecting phosphite-induced priming in *Arabidopsis* infected with *Hyaloperonospora arabidopsidis*. Plant Physiol. 2012;159: 286–298. 10.1104/pp.112.194647 22408091 PMC3375965

[43] Tsuda K, Somssich IE. Transcriptional networks in plant immunity. New Phytol. 2015;206: 932–947. 10.1111/nph.13286 25623163

[44] Checker VG, Kushwaha HR, Kumari P, Yadav S. Role of phytohormones in plant defense: Signaling and cross talk. Mol Asp Plant-Pathogen Interact. 2018; 159–184. 10.1007/978-981-10-7371-7_7

