# Supplementary material for: Identification of potassium phosphite responsive miRNAs and their targets in potato
Source: PLoS One. 2019 Sep 12;14(9):e0222346. doi: 10.1371/journal.pone.0222346 (PMC6742386; doi:10.1371/journal.pone.0222346)
Supplement: S1 Table — (DOC) [file pone.0222346.s001.doc]

**Table S1. Sequences of stem-loop, miRNA specific, Universal primers, and targets forward and reverse primers.**

| **miRNA** | **Stem-Loop RT primer** | **qPCR primer** | **Target accession** | **Description** | **qPCR forward primer** | **qPCR reverse primer** |
| --- | --- | --- | --- | --- | --- | --- |
| **stu-miR482a-3p** | GTCTCCTCTGGTGCAGGGTCCGAGGTATTCGCACCAGAGGAGACTAGGAA | GGCGGTTTCCAATTCCACCCA | PGSC0003DMT400012486 | Resistance protein PSH-RGH7 | GATGCACCAAGGGAAAGAAA | CCAGGCAACAAATCCTCATT |
| **stu-miR482c** | GTCTCCTCTGGTGCAGGGTCCGAGGTATTCGCACCAGAGGAGACTTGGCA | GGCGGTTTCCTATTCCACCCA | PGSC0003DMT400053047 | Tir-nbs-lrr resistance protein | GGCAAAACACAAGGAACGAT | CAGTCAGTGCTGCTTTCCAA |
| **stu-miR166b** | GTCTCCTCTGGTGCAGGGTCCGAGGTATTCGCACCAGAGGAGACGAGGAA | GGCGGTCGGACCAGGCTTCAT | PGSC0003DMT400074934 | BZIP domain class transcription factor | CCTTTTTGGGTGTTGAGGAA | GACGACGGAGTGAACTAGGC |
| **stu-miR171a-5p** | GTCTCCTCTGGTGCAGGGTCCGAGGTATTCGCACCAGAGGAGACTCTGAG | GGCGGTATTGGCCTGGTTCAC | PGSC0003DMT400066685 | Methylketone synthase Ib | GGTTCCGCTAATGGAGTTCA | GGCATCAGAGCAGTGACAAA |
| **stu-miR530_L-2R+2** | GTCTCCTCTGGTGCAGGGTCCGAGGTATTCGCACCAGAGGAGACTAAGGT | GGCGGTGCATTTGCACCTGCA | PGSC0003DMT400002883 | Zinc knuckle (CCHC-type) family protein | CGCTGTAGGTGCAGGTGTAA | GCCACTCTCAGCACATTTGA |
| **stu-MIR7985-p5** | GTCGTATCCAGTGCAGGGTCCGAGGTATTCGCACTGGATACGACGACGAG | GTATACTTTGCCTTCTGGGTC | PGSC0003DMT400062931 | Spotted leaf protein | GTCAATAGTGGTGCGGAGGT | CTTCTTTGCTTGGCTGGTTC |
| **PC-5p-1305_1307** | GTCTCCTCTGGTGCAGGGTCCGAGGTATTCGCACCAGAGGAGACTCTCAA | GGCGGCTTTGTGAAATGACTT | PGSC0003DMT400027717 | F-box protein family | TTTGACTTGCAGGCACTGAC | AAACAGGGTGGTGGACAAAG |
| **Universal reverse primer** | TGGTGCAGGGTCCGAGGTATT |  |  |  |  |  |
